# Identification of virulence associated milRNAs and their bidirectional targets in *Rhizoctonia solani* and maize during infection

**DOI:** 10.1186/s12870-021-02930-w

**Published:** 2021-03-26

**Authors:** Hongxu Meng, Shaoli Wang, Wei Yang, Xinhua Ding, Ning Li, Zhaohui Chu, Xiaoming Li

**Affiliations:** 1 State Key Laboratory of Crop Biology, College of Agronomy, Shandong Agricultural University, Tai’an, 271018 Shandong People’s Republic of China; 2 Yantai Academy of Agricultural Sciences, Yan’tai, 265500 Shandong People’s Republic of China; 3 Key Laboratory of Quality Improvement of Agricultural Products of Zhejiang Province, School of Agriculture and Food Science, Zhejiang A&F University, Lin’an, Hangzhou, 311300 Zhejiang China; 4 Shandong Provincial Key Laboratory for Biology of Vegetable Diseases and Insect Pests, College of Plant Protection, Shandong Agricultural University, Tai’an, 271018 Shandong People’s Republic of China

**Keywords:** Banded leaf and sheath blight, Illumina sequencing, Pathogenesis, Small RNA, Soil-born disease, *Zea mays*

## Abstract

**Background:**

Anastomosis group 1 IA (AG1-IA) of *Rhizoctonia solani* is the major agent of banded leaf and sheath blight (BLSB) disease that causes severe yield loss in many worldwide crops. MicroRNAs (miRNAs) are ~ 22 nt non-coding RNAs that negatively regulate gene expression levels by mRNA degradation or translation inhibition. A better understanding of miRNA function during AG1-IA infection can expedite to elucidate the molecular mechanisms of fungi-host interactions.

**Results:**

In this study, we sequenced three small RNA libraries obtained from the mycelium of AG1-IA isolate, non-infected maize sheath and mixed maize sheath 3 days after inoculation. In total, 137 conserved and 34 novel microRNA-like small RNAs (milRNAs) were identified from the pathogen. Among these, one novel and 17 conserved milRNAs were identified as potential virulence-associated (VA) milRNAs. Subsequently, the prediction of target genes for these milRNAs was performed in both AG1-IA and maize, while functional annotation of these targets suggested a link to pathogenesis-related biological processes. Further, expression patterns of these virulence-associated milRNAs demonstrated that theyparticipate in the virulence of AG1-IA. Finally, regulation of one maize targeting gene, *GRMZM2G412674* for Rhi-milRNA-9829-5p, was validated by dual-luciferase assay and identified to play a positive role in BLSB resistance in two maize mutants. These results suggest the global differentially expressed milRNAs of *R. solani* AG1-IA that participate in the regulation of target genes in both AG1-IA and maize to reinforce its pathogenicity.

**Conclusions:**

Our data have provided a comprehensive overview of the VA-milRNAs of *R. solani* and identified that they are probably the virulence factors by directly interfered in host targeting genes. These results offer new insights on the molecular mechanisms of *R.solani*-maize interactions during the process of infection.

**Supplementary Information:**

The online version contains supplementary material available at 10.1186/s12870-021-02930-w.

## Background

Banded leaf and sheath blight (BLSB) is one of the most devastating disease of plants, causing severe yield losses in maize worldwide. *Rhizoctonia solani* belongs to the soil-borne Basidiomycete fungus, which is the major agent of BLSB [1]. There are 14 anastomosis groups, termed AG1 to AG13 and AGBI based on their hyphal anastomosis and physiological/biochemical characteristics in *R.solani*. Among them, the anastomosis group 1 IA (AG1-IA) is the primary pathogen that causes BLSB or brown patches on more than 27 families of monocots and dicots [2]. As the causal agent of BLSB in maize, AG1-IA often exists as an asexual fungus. Occasionally, sexual structures from its teleomorph (*Thanatephorus cucumeris*) are observed in fields [3]. Vegetative mycelia and sclerotia are the source of primary infection for *R.solani* [4]. Usually, the pathogen initially infects maize at the first and second leaf sheath above the ground and then spreads upward to the ear, leading to serious yield losses [5].

To date, genetic and molecular studies of resistance to the pathogen have been reported in diverse crops [6, 7]. In maize, it is primarily controlled by quantitative disease resistance [8]. Three significant quantitative trait loci (QTLs) located on chromosomes 2, 6 and 10 confer resistance to BLSB [5, 9]. Recently, an F-box-like protein, ZmFBL41, was identified to regulate BLSB resistance in maize by targeting secondary metabolism of lignin [10]. Interestingly, a ubiquitin-protein ligase expression was induced dependent on *R.solani*-specific *cis*-elements in its promoter [11]. To explore defense-related genes, large scale sequencing technology has been used to identify hundreds of pathogen-induced genes [12, 13]. A number of catalytic enzymes, including chitinase, glucanase and phenylanine ammonia lyase, have been identified in response to the pathogen [14–16], as well as several pathogenesis-related (*PR*) genes and transcription factors involved in potential defense pathways [13, 17, 18]. For the pathogen, whole genome sequences of strains of the rice-infecting pathogen *R. solani* AG1-IA were generated in 2013 using Illumina technology, which provided new insights into the pathogenic mechanisms of AG1-IA and the molecular basis of pathogen-host interactions [19].

MiRNAs comprise a class of endogenous, single-stranded and non-coding small RNAs that are usually 21 or 22 nucleotides (nt) in length. Although the first miRNA gene *lin-4*, was characterized from *Caenorhabditis elegans* in 1993 [20], miRNAs were not validated as a distinct class of biological regulators until 2000 [21]. To date, a fast growth rate of studies has demonstrated that miRNAs play important roles in various biological processes by regulating expression levels of their target genes [22]. In plants, most miRNA genes are transcribed by RNA polymeraseII into primary miRNAs (pri-miRNAs), which are primarily cleaved by the enzyme dicer-like 1 (DCL1) to generate a stem-loop structure known as the precursor miRNA (pre-miRNA). Pre-miRNAs are further processed to produce a miRNA/miRNA duplex by DCL1, and then miRNAs are incorporated into the RNA-induced silencing complex (RISC) for target repression at the posttranscriptional level via the activity of ARGONAUTE (AGO) proteins, while miRNAs* are usually degraded [23]. Although miRNAs have been studied intensively in both plants and animals, miRNA pathways remains poorly understood and even controversial in most fungal species. Recently, small RNAs that are distinct from those in animals and plants have been identified in several fungi via the application of deep sequencing. For instance, qiRNAs were described in *Neurospora crassa* as a new type small interfering RNA induced by DNA damage and requiring quelling deficient-1 (QDE-1, an RNA-dependent RNA polymerase), QDE-3 (a Werner and Bloom RecQ DNA helicase homologue) and dicer proteins [24]. However, fungi were thought not to have miRNAs until the discovery of miRNA-like small RNAs (milRNAs) in *N.crassa*. Surprisingly, at least four different mechanisms that use a distinct combination of factors were discovered to produce milRNAs. Meanwhile, dicer-independent small interfering RNAs (disiRNAs) with a size of 21 or 22 nt were also recognized in *Neurospora* [25]. Afterwards, a number of milRNAs was identified in fungi. For example, researchers reported fifteen milRNAs in *Metarhizium anisopliae* that may regulate processes of mycelium growth and conidiogenesis [26]. In the plant pathogenic fungus *Sclerotinia sclerotiorum*, two milRNAs and 42 milRNA candidates were identified by high-throughput sequencing [27], while milRNAs and their hairpin precursors were observed in the fungus *Cryptococcus neoformans* using bioinformatics and northern blotting approaches [28]. In addition, it was reported that milRNAs recruit different components of the RNA silencing protein apparatus to generate small RNAs that vary from 19 to 25 nt in several filamentous fungi [29, 30]. These differences indicate that fungal milRNA production may have evolved independently from that in plants and animals. Interestingly, small RNAs of *Botrytis cinerea* are able to hijack the host RNA interference (RNAi) machinery by binding to the AGO1 protein to suppress plant immunity, demonstrating that a trans-kingdom RNAi is a virulence mechanism of the pathogen in fungus-plant interactions [31]. Since then, a growing number of studies suggests that fungi small RNAs can transfer into plants and exert bidirectional functions for their own benefit [32]. For *R.solani*, Lin et al. [33] identified 177 milRNAs, including 15 pathogenic novel milRNAs, after sequencing the six small RNA libraries derived from the mixed RNA of hyphal and rice leaves during different infection periods.

To further understand the molecular mechanism of host-pathogen interactions, we applied small RNA high-throughput sequencing to identify virulence-associated milRNAs in Maize*r. solani* AG1-IA. Further, the regulatory roles of fungal milRNAs and their maize target genes were investigated by real-time PCR and a dual-luciferase assay. Moreover, we validated one maize target gene which act as a positive regulator of BLSB using two EMS mutants. Overall, we aimed to elucidate the small RNA transcriptome of AG1-IA in maize to understand the function of fungal pathogenic small RNAs.

## Results

### High-throughput sequencing of small RNAs from AG1-IA at infection stages

**Fig. 1:**
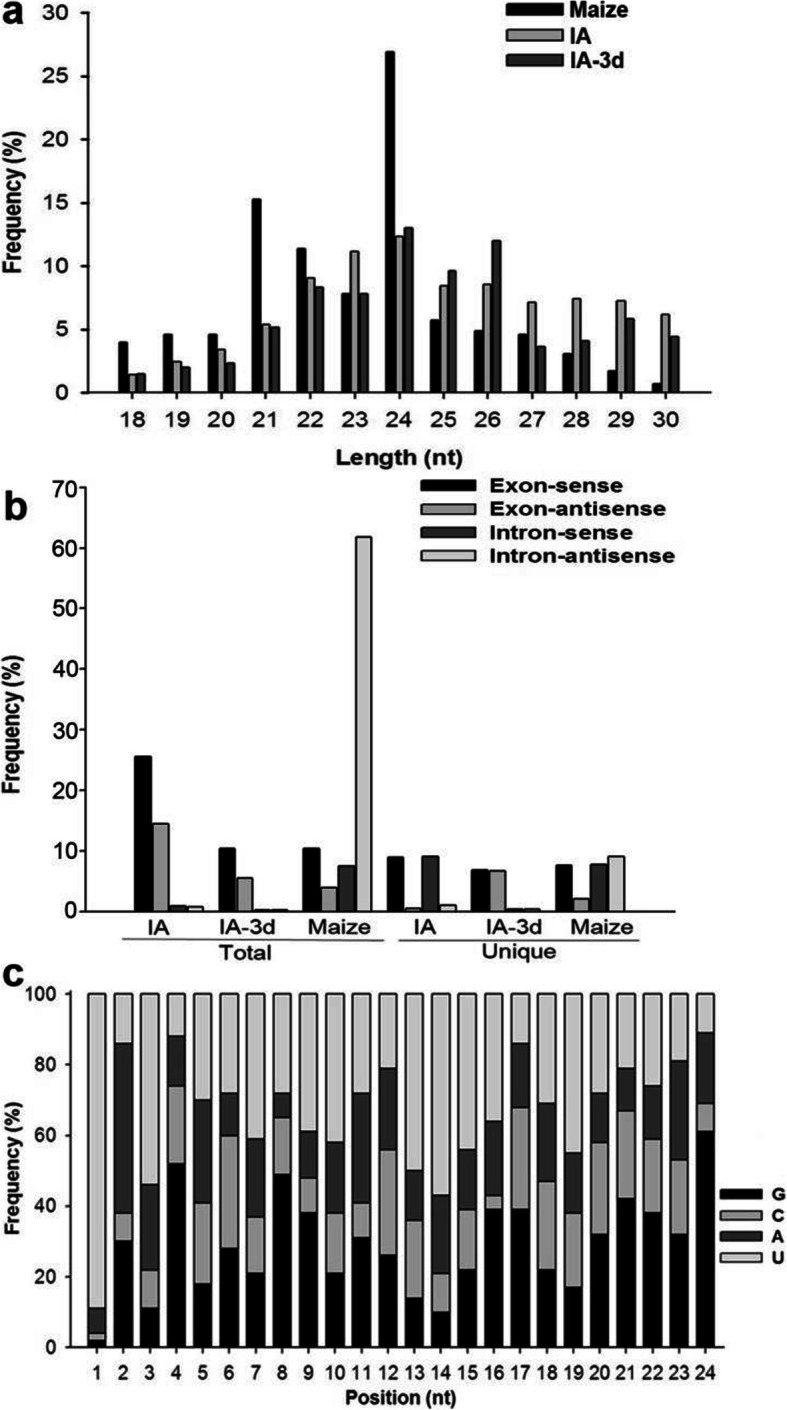
Characterization of small RNAs in AG1-IA and maize. (**a**) Length distribution of small RNAs identified from IA, IA-3d and maize. (**b**) Summary of the percentage of reads mapping to exons and introns. (**c**) Nucleotide bias at each position of milRNA candidates

To profile small RNAs expressed in AG1-IA and to identify milRNAs potentially involved in fungus-plant interactions, we sequenced three small RNA libraries from AG1-IA mycelium (IA), AG1-IA infected maize sheath three days after inoculation (IA-3d) and maize sheath (Maize) using Illumina technology. Ultimately, 14,358,708, 11,012,296 and 14,342,801 raw reads were obtained from IA, IA-3d and Maize, respectively. After removal of adaptors, contaminants and low-quality sequences, 14,345,211, 10,976,072 and 14,304,313 high-quality clean reads with sizes of 18–30 nt were generated from IA, IA-3d and Maize. Among these reads, 19–25 nt small RNAs comprised the major proportion (Fig. 1a).

Clean reads of IA and IA-3d were aligned against the whole genome sequence of *R. solani* AG1-IA using SOAP, revealing 13,929,970 and 10,611,599 total reads corresponding to 2,215,557 and 1,681,014 unique reads, respectively, that were perfectly matched with the fungal genomic sequences. Also, we analyzed the reads from IA and IA-3d libraries with maize genome and found that 9,120,804 and 8,866,147 total reads corresponding to 1,756,096 and 858,176 unique reads from IA and IA-3dwere mapped to the maize genome, respectively. Meanwhile, the clean reads of Maize were mapped to the B73(B73 RefGen_3v, released 5b+) and fungal genome, respectively. Finally, 11,698,169 total reads representing 3,414,618 unique reads matching the maize genome were obtained, while 7,325,577 total reads representing 599,163 unique reads were mapped to the fungal genome (Table 1). Subsequently, we aligned all of the reads against the exon and intron sequences of their respective genome. It is indicated that exon-sense regions are the major source for the reads production in IA and IA-3d, accounting for 25.51 and 10.38%, respectively. However, most of the maize sequences (61.85%) were generated from intron-antisense regions (Fig. 1b). As the small RNAs were non-coding RNAs, the reads mapping to exons and introns would be excluded in the next analysis. Meanwhile, based on a BLASTN search against the Rfam database, sequences representing snRNA, tRNA, rRNA and other small RNAs were identified (Table 1). Unknown reads, designated as unannotated small RNAs, provided an opportunity to identify novel milRNAs in both AG1-IA and maize. Analysis of these small RNAs also suggested a bias for U enrichment and C suppression at the first nucleotide position (Fig. 1c), which is consistent with other observations in fungi [25, 30, 34].

**Table 1:** Summary of total genome-matched sequences identified from three libraries

	Maize		IA		IA-3d	
	Unique	Total	Unique	Total	Unique	Total
Total reads^a^	599,163	7,325,577	858,176	8,866,147	1,756,096	9,120,804
Total reads^b^	3,414,618	11,698,169	2,215,557	13,929,970	1,681,014	10,611,599
snRNA	7854	29,338	1579	17,643	6338	22,109
tRNA	25,316	340,561	35,689	1,505,326	36,319	1,116,975
rRNA	215,474	5,142,028	183,071	5,555,101	175,294	2,881,964
snoRNA	4289	14,272	774	5152	5107	21,046
miRNA	11,728	1,327,871	74,129	534,082	47,280	607,097
Other small RNAs	935,240	701,340	273,045	785,262	144,535	243,513
Unannotated	2,214,717	4,142,759	1,647,270	5,526,804	1,266,141	5,718,895

^a^ Refer to the reads number of Maize mapped to the fungal genome and the reads number of IA and IA-3d mapped to the maize genome

^b^ Refer to the reads number of Maize mapped to maize genome and the reads number of IA and IA-3d mapped to the fungal genome

### Identification of conserved and novel milRNAs in AG1-IA

To identify milRNAs in AG1-IA, a modified method for fungal milRNA prediction by MIREAP and miRDeep2 software was used [33]. Finally, 137 conserved milRNAs with a total of 88,161 transcripts per million (TPM) were identified in the IA library (Supplementary Table S1). Among the 137 conserved milRNAs, 29 had relatively high expression greater than 1000 TPM, suggesting their abundance in fungi. In addition, 34 novel milRNAs with a total of 406 TPM were found in the IA library and 10 novel milRNAs exhibited expression levels more than 10 TPM (Supplementary Table S1). All of the precursors of these milRNAs have a typical hairpin structure, and the secondary structures of 10 randomly selected milRNA precursors are shown in Fig. S1.

### Target gene prediction of milRNAs in *R. solani* AG1-IA

To demonstrate the potential roles of the identified milRNAs, target genes in the AG1-IA genome were predicted using psRNATarget. Therefore, 661 target genes of 150 milRNAs consisting of 119 conserved and 31 novel milRNAs were identified (Supplementary Table S2). Among the 150 milRNAs, 15 (10.00%) were predicted to have at least 10 target genes, while only 11 (7.33%) milRNAs had a single predicted target. Interestingly, 579 (87.59%) of the 661 targets were predicted to be regulated by a single milRNA with a specific targeting site while 80 (12.10%) genes were predicted to be regulated by at least two milRNAs or by a single milRNA with several different sites, such as Rhi-milR-7566 and AG1IA_07036. Target genes were not found for 21 milRNAs, likely due to mismatches between the AG1-IA genome and milRNAs or a lack of target gene annotation in the genome.

To investigate the potential roles of milRNAs in pathogenicity, functional annotation of the 661 target genes was performed. Totally, 473 genes were annotated in the AG1-IA genome, including nine ABC transporters, 15 cytochrome P450s (CYPs), 54 secreted proteins and other factors (Supplementary Table S3, S4). AG1IA_08015, regulated by Rhi-milR-1203, was classified into the fungal ABC transporter G family, which may contribute to pleiotropic drug resistance and is associated with translocation of phospholipid molecules [35–37]. Additionally, six transporters of AG1-IA categorized in the Transporter Classification Database (TCDB) [38] were annotated (Supplementary Table S4). Interestingly, a total of 54 secreted genes were predicted to be targets of milRNAs (Supplementary Table S3), indicating that milRNAs may act as negative regulators of secreted genes during fungal infection. Particularly, as candidate effectors, four cysteine-rich proteins were characterized among the 54 secreted proteins.

Among the other candidate milRNA targets, 23 of them were categorized as CAZymes including six glycoside hydrolase (GHs) and 13 glycosyl-transferase (GTs), which are involved in the biosynthesis and degradation of glycogen in fungi [39–41]. AG1IA_08946, predicted as the target gene of Rhi-milR-81, was annotated to encode a putative cellulase that may contribute to virulence of fungi through degradation of the plant cell wall [42]. Fourteen Skp1-Cul1-F-box (SCF) genes were identified by alignments to known SCFs in fungi (Supplementary Table S4), while AG1IA_01201 regulated by Rhi-milR-1418-5p was predicted to encode an SCF subunit. The results indicate that milRNAs are potentially required for virulence of AG1-IA by targeting the CAZymes, the SCF complex, MAPK and calcium signaling pathways (Supplementary Table S4). Interestingly, different target genes regulated by Rhi-milR-1418-5p were involved in both MAPK and calcium pathways simultaneously, indicating multiple regulation points of biological processes by milRNAs in fungus.

To define the potential pathogenicity of the predicted genes, the 473 target genes were assigned to a pathogen-host interaction (PHI) database [43], resulting in 37 genes regulated by 42 milRNAs that were characterized as PHIs (Supplementary Table S4). We found that 23 PHIs were related to reduced virulence, while three PHIs were related to increased virulence. Moreover, functional annotation of the PHIs indicated the inclusion of eight CAZymes, four ABC transporters and two secreted proteins, suggesting that milRNAs are likely to play important roles in the regulation of these target genes with respect to pathogenicity.

To gain a better understanding of their functional roles in AG1-IA, Gene Ontology (GO) analysis was performed on the predicted target genes. Totally, 214 genes had assignments of GO molecular function, biological processes and cellular components categories. These genes showed a strong affinity for binding activity, signal transduction factors and hydrolase activity, all of which are typically important during infection stages (Supplementary Table S5). These findings indicate that milRNAs may have multiple molecular functions or mediate diverse biological processes through their targets during infection.

### Identification of virulence associated milRNAs and target genes during infection

**Fig. 2:**
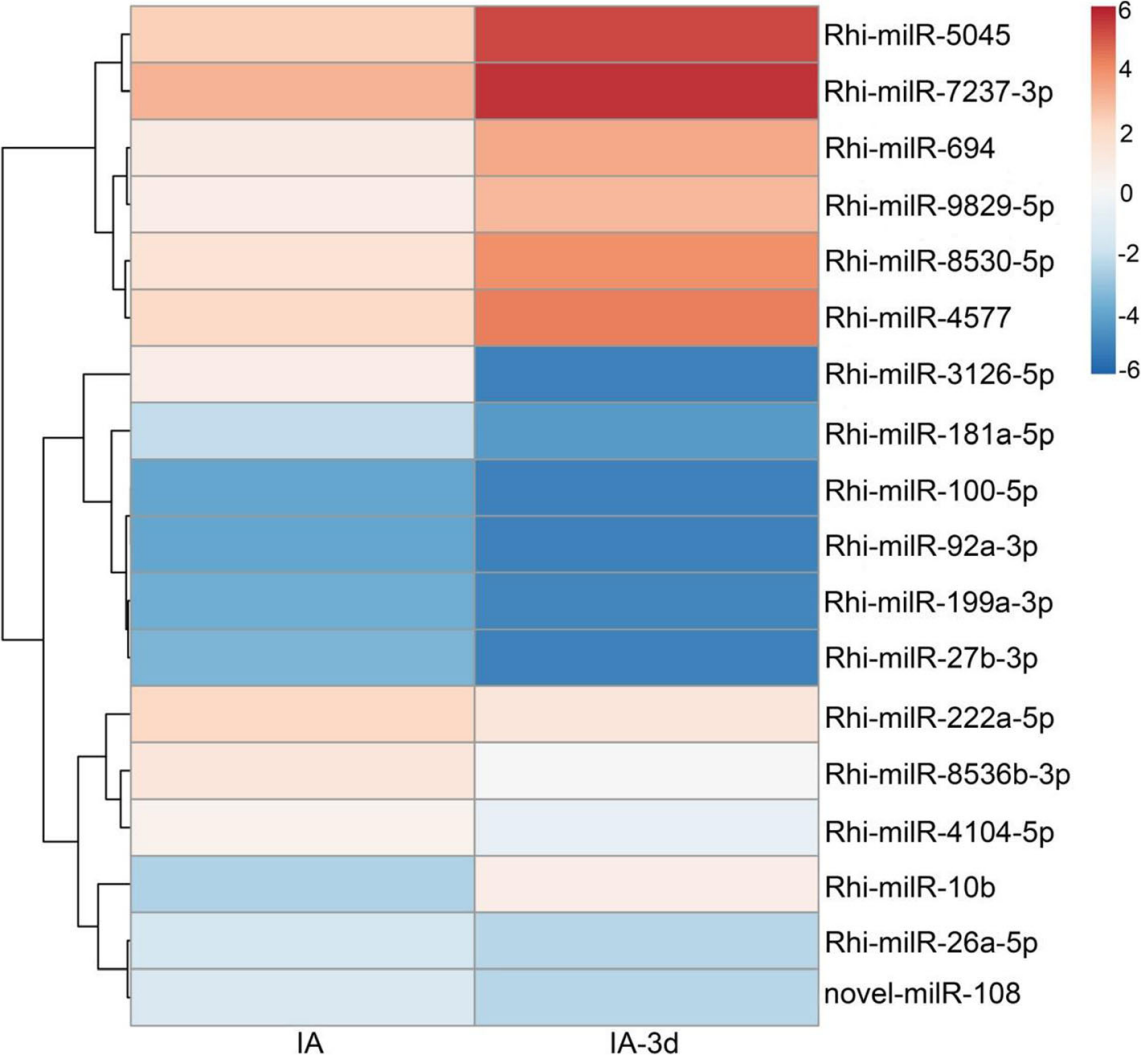
The expression pattern of VA-milRNAs during infection process

Identification of virulence-associated milRNAs will facilitate our understanding of the molecular regulation of AG1-IA during infection. For this purpose, normalized expression levels of milRNAs greater than 2.5 TPM in both IA and IA-3d libraries were selected and compared. Finally, milRNAs with fold-changes greater than 1.5 (log_2_ ratio) and *P*-values less than 0.05 (Chi-squared test) were termed putative virulence associated milRNAs (VA-milRNAs) and chosen for further analysis. As shown in Figs. 2, 17 conserved milRNAs and the novel-milR-108 were found to be VA-milRNAs. Among 18 VA-milRNAs, seven were upregulated, while 10 conserved milRNAs and the novel-milR-108 were downregulated (Fig. 2; Supplementary Table S6) in IA-3d compared to IA.

To demonstrate the potential roles of VA-milRNAs during pathogenicity, we examined the target genes of these milRNAs previously predicted in AG1-IA. Totally, 89 target genes regulated by these 18 VA-milRNAs were identified (Supplementary Table S7). Among these, only AG1IA_02109 was targeted by two VA-milRNAs (Rhi-milR-3126-5p and Rhi-milR-8530-5p) at different sites, while the other genes were regulated by a single VA-milRNA.

Subsequently, functional annotation of these 89 target genes was performed to reveal their potential roles. In total, 58 of the 89 target genes have been functionally annotated in the *R. solani* genome (Supplementary Table S8). Among them, we identified four ABC transporters, including AG1IA_2109, AG1IA_3327, AG1IA_3597 and AG1IA_6835, three CYPs, three CAZymes, three SCFs and six secreted genes containing two cysteine-rich genes. In addition, AG1IA_04862, regulated by Rhi-milR-3126-5p, was categorized in both the MAPK pathway and as a CAZyme member, suggesting its diverse functions during infection.

To explore the potential roles of these VA-milRNAs and their targets, the 58 genes were assigned to the PHI database. Nine genes regulated by ten milRNAs were identified as PHIs (Supplementary Table S8). AG1IA_05348 and AG1IA_05617 were relevant to loss of pathogenicity, while the other seven genes were related to reduced virulence. Furthermore, functional annotation of the nine PHIs showed that they belong to ABC transporter, glycosyl-transferase, cadmium ion transporter, isocitratelyase and DNA repair proteins. It is implied that the ten milRNAs may participate in pathogenesis by negatively regulating their PHI targets. GO analysis was also performed to understand the potential roles of the 58 targets containing nine PHIs (Supplementary Table S9). The results demonstrated that these targets are involved in the cell cycle, metabolic, microtubule-based and signal transduction processes. The above results indicated that the nine VA-milRNAs targeting PHIs are likely engaged in different molecular and biological processes that affect pathogenicity.

### Expression pattern of milRNAs and their target genes in AG1-IA

**Fig. 3:**
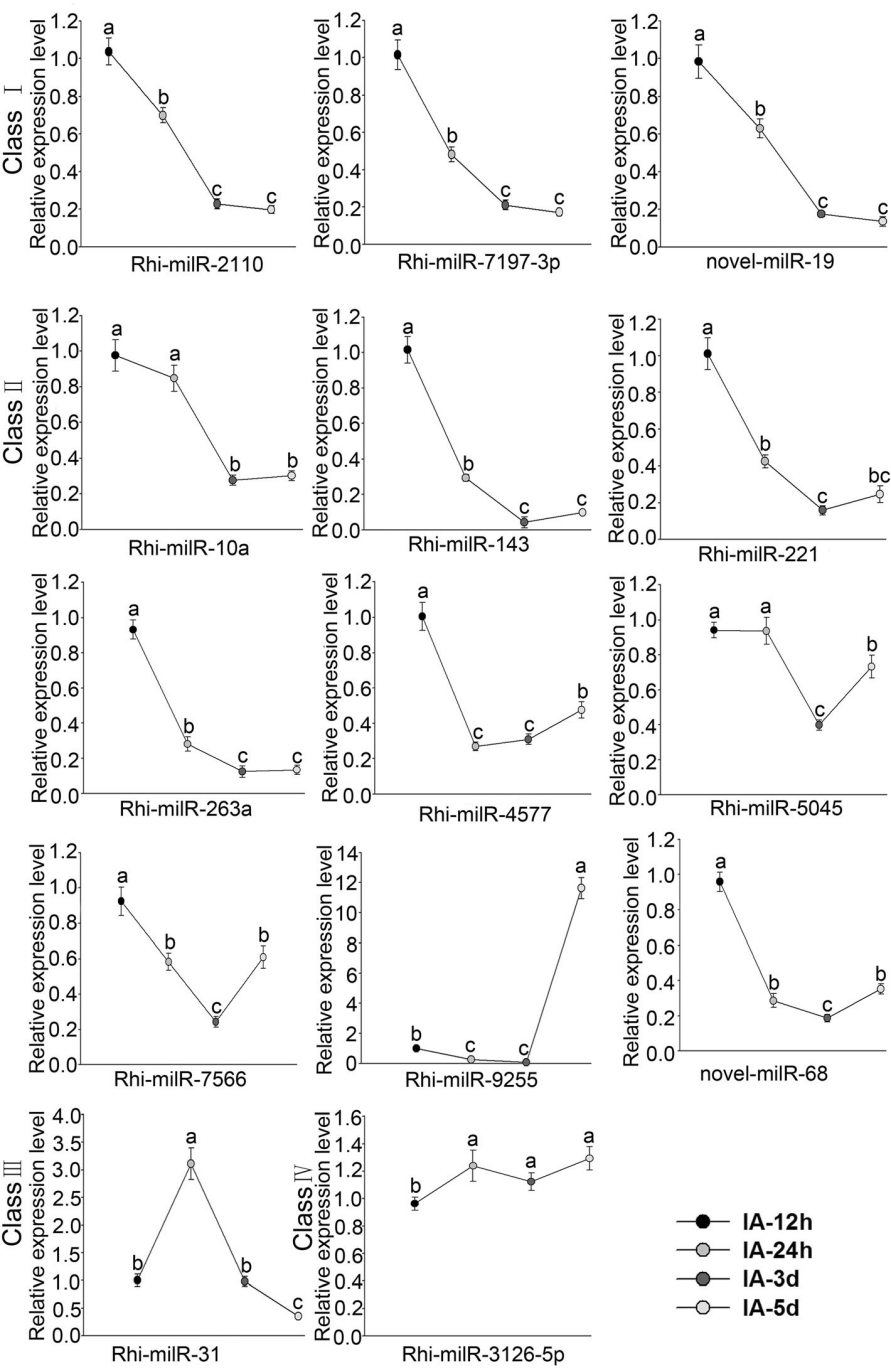
Relative expression levels of selected milRNAs during *R. solani* culture processing stem-loop real-time PCR. IA-12 h, IA-24 h, IA-3d, and IA-5d indicate the time at 12 h, 24 h, 3d, and 5d after medium culture, respectively. The bars represent the mean ± SD. The different lowercase letters indicate significant differences with *P-*value < 0.05

To determine expression levels of milRNAs during culture process of *R. solani*, 14 milRNAs were randomly selected for real-time RT-PCR analysis from 171 identified milRNAs. As shown in Fig. 2, the milRNAs were classified into four categories due to their expression pattern. The first, containing Rhi-milR-2110, Rhi-milR-7197-3p and novel-19, was “gradually decreased” milRNAs during the fungi culture process. Meanwhile, expression levels of nine milRNAs, including Rhi-milR-4577 and Rhi-milR-5045, showed a trend of “decreased at first and then increased”, while Rhi-milR-31 displayed an expression pattern of “rise first and then fall”. Expression of Rhi-milR-3126-5p was a little different from the other three types and exhibited a trend of “increased then decreased and then increased again” (Fig. 3).

Among the 14 milRNA targeting genes, AG1IA_03824 and AG1IA_07031 encoded a cell division cycle (CDC) protein and an elongation factor, respectively, and were predicted to be the targets of Rhi-milR-7197-3p. Meanwhile, AG1IA_00782, encoding a WD-repeat containing protein, was predicted to be regulated by Rhi-milR-2110. These results demonstrate that Rhi-milR-7197-3p and Rhi-milR-2110 may participate in the propagation of *R. solani* in maize by regulating their targets. In addition, the novel milR-19, which targeted AG1IA_00523,encoding a glycosyl-transferase was decreased during infection. Generally, the decrease of milRNA abundances was correlated with the increase of target genes, suggesting that propagation of AG1-IA requires regulation by multiple milRNAs and diverse signal transduction pathways. These results suggest that these milRNAs exist in AG1-IA and participate in pathogenic propagation in maize.

**Fig. 4:**
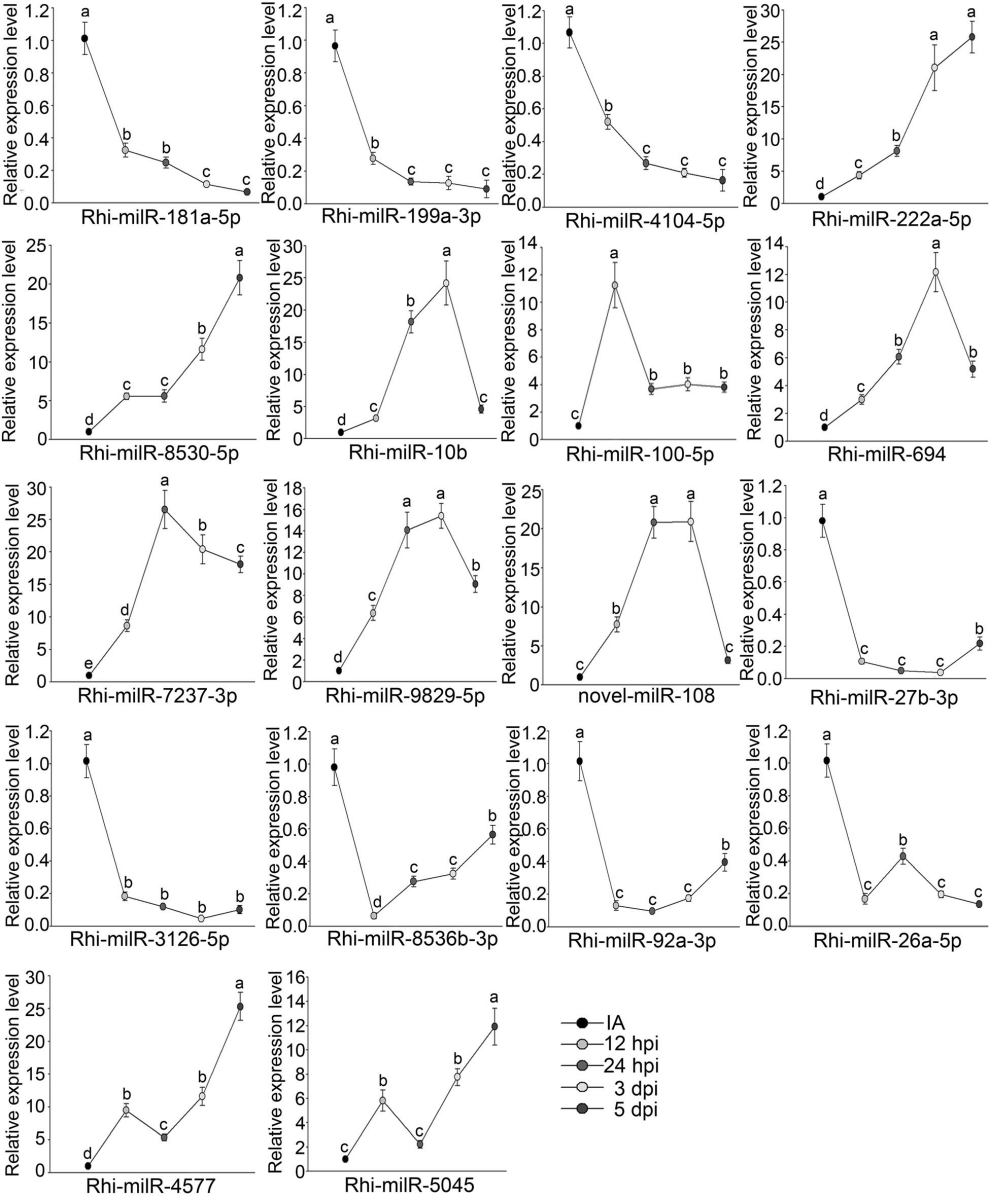
Validation of VA milRNAs in maize at different infection stages using real-time PCR. IA, 12 hpi, 24 hpi, 3 dpi, and 5 dpi indicate 0 h, 12 h, 24 h, 3d, and 5d post inoculation, respectively. The bars represent the mean ± SD. The different lowercase letters indicate significant differences with *P-*value < 0.05

To validate expression patterns of the above18 VA-milRNAs, we performed real-time RT-PCR. Results showed that expression patterns of 15 VA-milRNAs were consistent with those detected by high-throughput sequencing, while Rhi-milR-100-5p, Rhi-milR-222a-5p and novel-milR-108 were slightly different (Fig. 4). Additionally, expression levels of VA-milRNAs during infection stages of 12 h,24 h and 5d were determined. As shown in Fig. 4, expression of several VA-milRNAs, including Rhi-milR-199a-3p and Rhi-milR-4104-5p,continuously decreased over 12 h, 24 h, 3d and 5d stages, suggesting upregulation of their targets and implicating these VA-milRNAs as having a role in pathogenicity. In addition, a few VA-milRNAs, such as Rhi-milR-222a-5p and Rhi-milR-8530-5p, were nearly constantly upregulated during infection, suggesting that expression of their target genes was suppressed and implying that these targets might negatively regulate virulence.

**Fig. 5:**
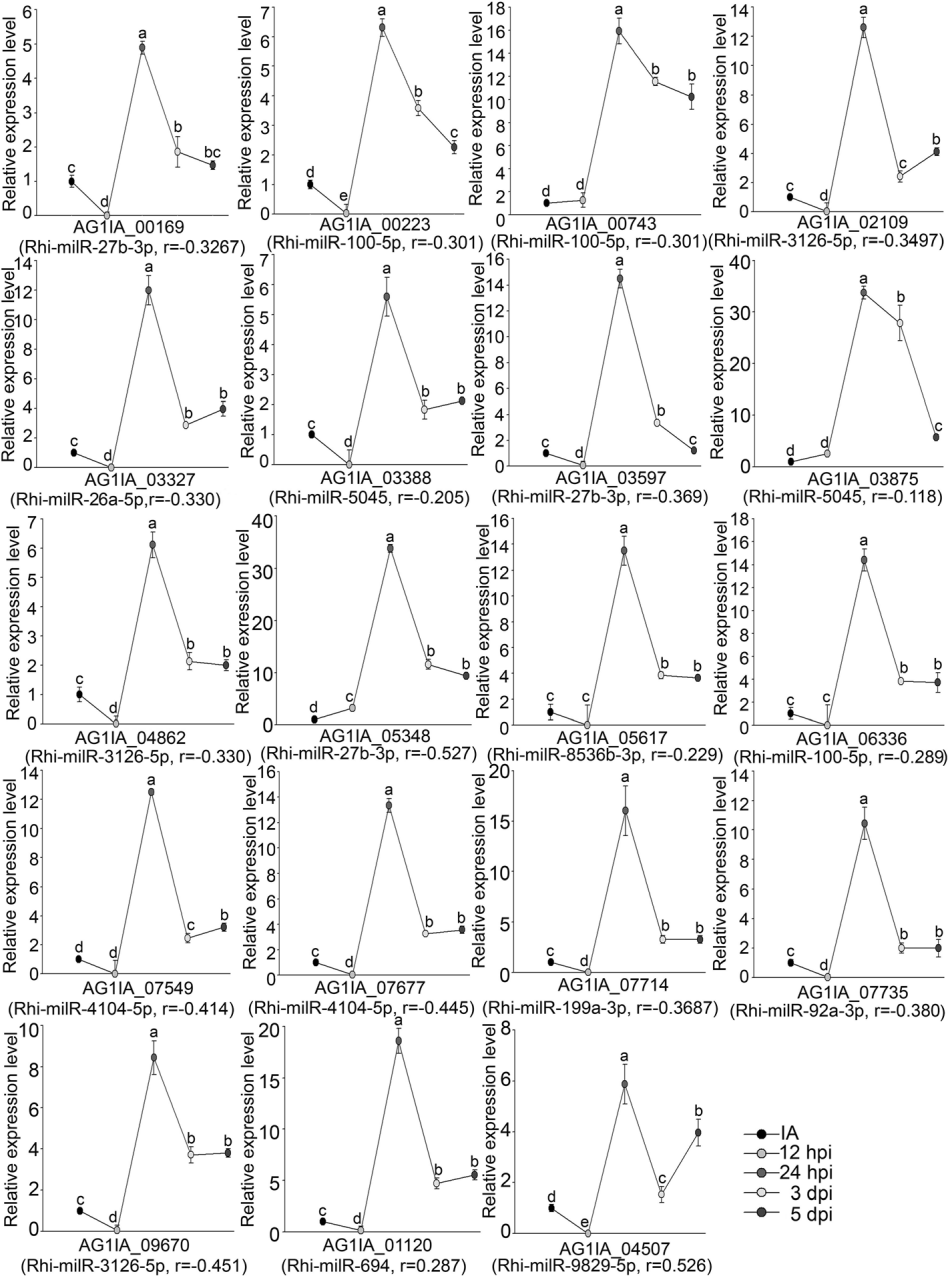
Expression patterns of selected candidate target genes in AG1-IA of VA milRNAs using real-time RT-PCR. IA, 12 hpi, 24 hpi, 3 dpi, and 5 dpi indicate 0 h, 12 h, 24 h, 3d, and 5d post inoculation, respectively. The bars represent the mean ± SD; r represents the correlation value between expression levels of milRNAs and their targets. The different lowercase letters indicate significant differences with *P-*value < 0.05

To determine the expression pattern of VA-milRNA targeted genes in AG1-IA, 19 candidates regulated by 12 milRNAs were examined by real-time RT-PCR. As shown in Fig. 5, all 19 genes exhibited their highest expression levels at 24 h after infection, indicating the intensive expression of virulence related genes at this stage. To assess whether these target genes were negatively regulated by milRNAs, correlation analysis between the expression levels of milRNAs and their target genes was performed. Among the 19 genes, expression levels of 12 target genes were moderate negatively correlated (− 0.6 < r ≤ − 0.3) with their milRNAs, while five genes displayed only weak negative correlations (− 0.3 < r ≤ 0) with their milRNAs. These results suggest that the target genes are negatively differentially regulated by VA-milRNAs. However, expression of AG1IA_01120 and AG1IA_04507 were positively correlated with expression levels of VA-milRNAs. This might be due to the target genes being regulated through translational repression by milRNAs or coregulated by other unknown factors.

### Predicting the targeted host genes for VA milRNAs

Small RNAs of pathogens also act as direct virulence factors that manipulate host target genes [31]. Therefore, we analyzed the putative targets of 18 VA-milRNAs in maize. Except for Rhi-milR-92a-3p and Rhi-milR-222a-5p, which have no putative target genes, the other 16 VA-milRNAs have been identified to target a total of 56 maize genes (Supplementary Table S10). Among them, Rhi-milR-3126-5p and Rhi-milR-5045 were revealed to target ten and eight genes, respectively. In contrast, a single gene in maize may also be targeted by different milRNAs. For instance, GRMZM2G009555 was matched by both Rhi-milR-4577 and Rhi-milR-3126-5p at distinct target sites.

To determine the function of VA-milRNAs in fungus-host interactions, functional annotation of their target genes in maize was performed. The results revealed that 28 genes had been annotated in the B73 genome database (Supplementary Table S11) as tightly associated with plant immunity. Among these genes, five transferases including DNA methyl-transferase, glutathione transferase and GlcNAc-1-p transferase, were characterized. The GRMZM2G064628 encoding Teosinte branched/Cycloidea/Proliferating cell factor 18 (TCP18), which is a class of plant-specific transcription factors involved in controlling the fate of plant organ growth and regulating part of hormone biosynthesis and signal transduction pathways [44, 45], was also identified to be a putative target of Rhi-milR-5045. Notably, GRMZM2G154449 encoding a Thaumatin-like protein (TLP), was predicted to be targeted by Rhi-milR-4104-5p. Previous studies have revealed that plant TLP is classified into the PR protein family 5 (PR5) and exhibits an antifungal property [46]. These results indicated that Rhi-milR-4104-5p may negatively regulate resistance of maize by targeting TLP genes. Furthermore, the putative target of Rhi-milR-7237-3p, GRMZM2G357399, encodes an ADP-ribosylation factor, which could induce expression of PR genes and resistance to fungal pathogens in tobacco [47]. In addition, the formin-like protein, WD-40 repeat family protein, transporter MRS2 and ATPase, were also identified among the target genes.

GO analysis was performed for these VA-milRNAs targets. Only four genes, consisting of AC148152.3, GRMZM2G025592, GRMZM2G033219 and GRMZM2G036720, were categorized with GO terms (Supplementary Table S11). The biological process terms were assigned to carbohydrate metabolic process (GO: 0005975), DNA methylation (GO: 0006306), chromatin assembly or disassembly (GO: 0006333), riboflavin biosynthetic process (GO: 0009231) and protein folding (GO: 0006457). Within the GO molecular function category, the terms hydrolase activity, DNA binding, chromatin binding and peptidyl-prolyl cis-trans isomerase activity were identified. It is inferred that fungal milRNAs may respond to signals from pathogen-host interactions by activating diverse biological processes and multiple molecular functions through regulating their host target genes during the infection process.

To further elucidate the mechanism of target genes associated with fungus-host interactions, the 1500 base pair (bp) upstream promoter sequences of the 58 targets in maize regulated by the VA-milRNAs were analyzed using PlantCARE (http://intra.psb.ugent.be:8080/PlantCARE). As listed in Supplementary Table S12, multiple *cis*-elements related to stress responsiveness were identified, such as the ABA-response elements (ABREs), P-box (gibberellin-responsive element) and MYB binding site (MBS) involved in drought inducibility. It is implied that the maize target of VA-miRNAs are more prone to select the stress responsive genes.

### Expression pattern of putative target genes for VA-milRNAs in maize

**Fig. 6:**
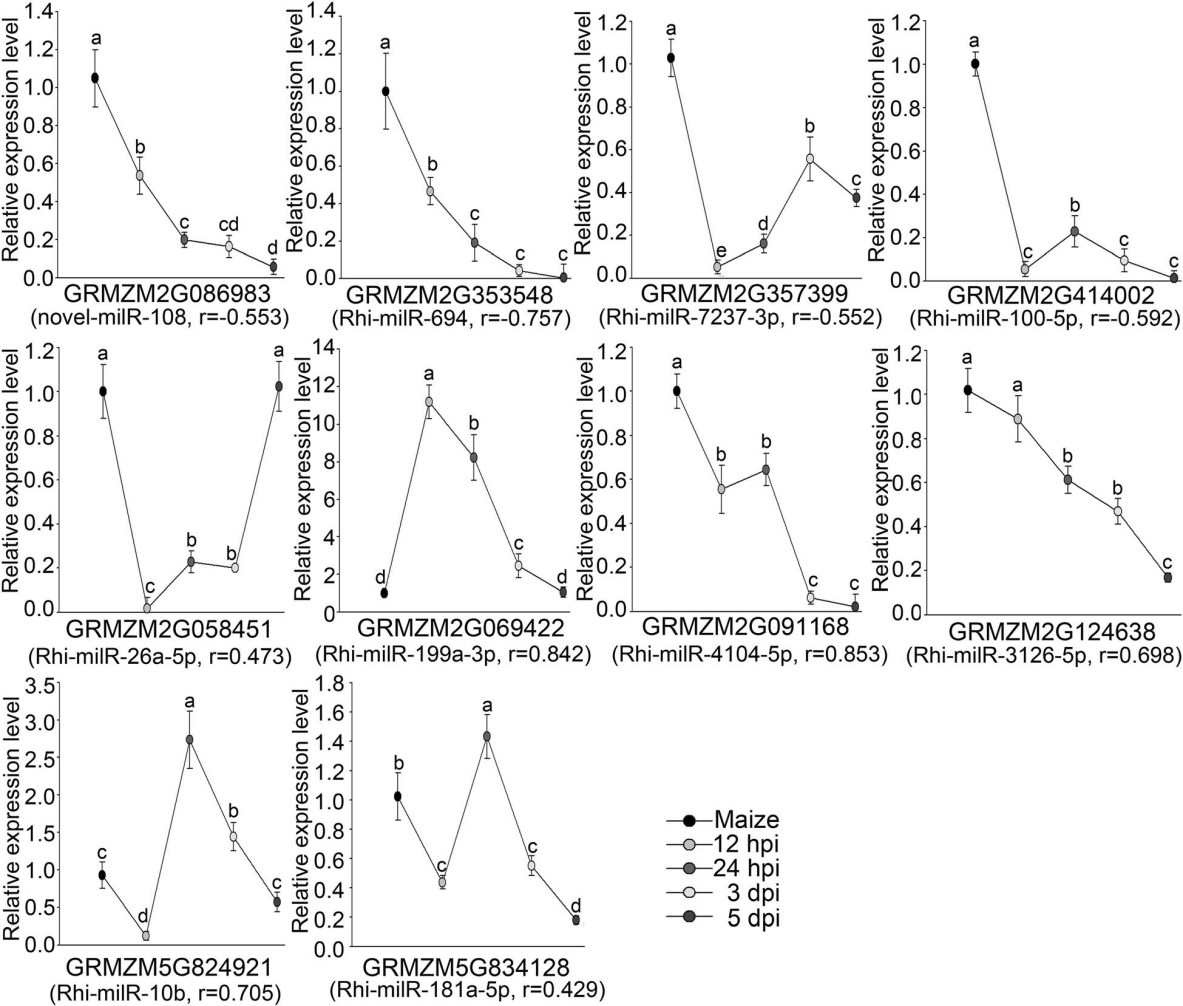
Expression patterns of selected predicted target genes in maize of VA milRNAs detected by real-time RT-PCR. Maize indicates the maize sheath; 12 hpi, 24 hpi, 3 dpi, and 5 dpi indicate 0 h, 12 h, 24 h, 3d, and 5d post inoculation, respectively. The bars represent the mean ± SD; r represents the correlation value between expression levels of milRNAs and their targets. The different lowercase letters indicate significant differences with *P-*value < 0.05

**Fig. 7:**
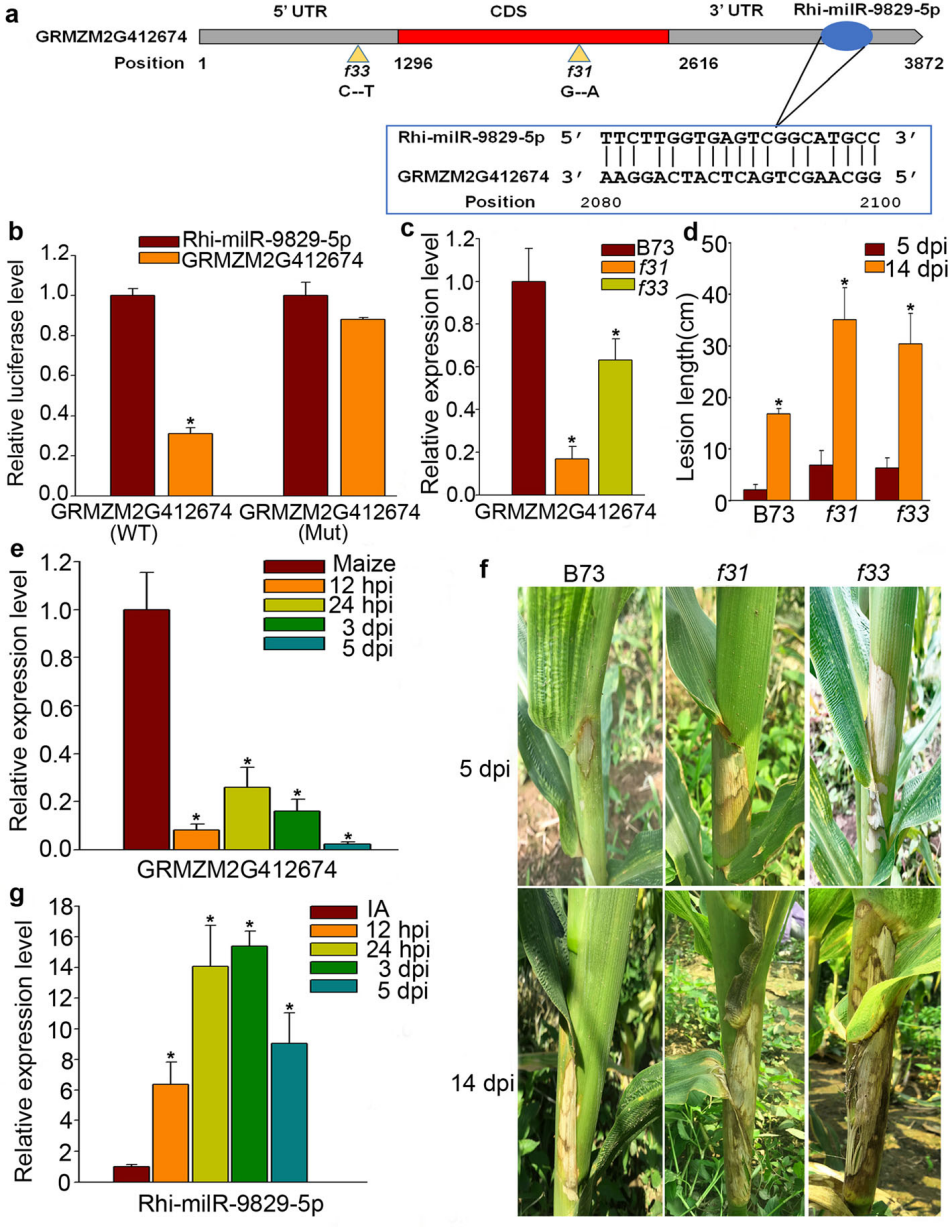
GRMZM2G412674 is attenuated expression by co-transforming with Rhi-milR-9829-5p and act as a positive regulator of maize BLSB. (**a**) The targeting sequence in the 3′ UTR region of GRMZM2G412674 by Rhi-milR-9829-5p and the mutation sites of *f31* and *f33* mutants. (**b**) Expression levels of GRMZM2G412674 assessed by the transient dual-luciferase assay. “GRMZM2G412674 (WT)” represents the predicted targeting sites, while “GRMZM2G412674 (Mut)” indicates the mutated target gene in the Rhi-milR-9829-5p binding sites. (**c**) Expression of GRMZM2G412674 in four-weeks B73, *f31* and *f33* lines using real-time RT-PCR. (**d**) Lesion length of the *f31* and *f33* mutant lines at 5 and 14 days after inoculation. (**e**) Expression patterns of GRMZM2G412674 detected by real-time RT-PCR. (**f**) Susceptible phenotype of the *f31* and *f33* mutant lines at 5 and 14 dpi. (**g**) Expression levels of Rhi-milR-9829-5p analyzed by real-time RT-PCR. 12 hpi, 24 hpi, 3 dpi, and 5 dpi indicate 0 h, 12 h, 24 h, 3d, and 5d post inoculation, respectively. The *bars* represent the mean ± SD. The asterisk indicates a significant difference with *P-*value < 0.05

To better understand the regulation of maize targets by *R.solani* VA-milRNAs, we selected 11 predicted target genes to analyze expression patterns through qRT-PCR (Figs.6 and 7e). Correlation analysis between expression levels of VA-milRNAs and their maize target genes revealed that *GRMZM2G353548* and *GRMZM2G412674* were strongly negatively correlated (− 0.8 < r ≤ − 0.6) with their VA-milRNAs, while three target genes *GRMZM2G086983*, *GRMZM2G357399* and *GRMZM2G414002* were moderately negatively correlated (− 0.6 < r ≤ − 0.3) with their VA-milRNAs. Thus, these maize target genes appear to be negatively regulated by the VA-milRNAs. However, expression levels of the other six genes were not negatively correlated with their milRNAs. Overall, these results demonstrated that part of the AG1-IA VA-milRNAs might regulate maize genes during fungi-host interactions.

### GRMZM2G412674 is a target of Rhi-milR9829-5p and involved in the response to *R. solani*

According to the bioinformatics prediction, *GRMZM2G412674* possesses a Rhi-milR9829-5p binding site located in the 3’UTR (Fig. 7a). To determine whether this target gene was negatively regulated by Rhi-milR9829-5p, a dual-luciferase (LUC) assay was performed in tobacco leaves. We found that luciferase activity of GRMZM2G412674 3’UTR was decreased in response to co-expression with Rhi-milR9829-5p, while the negative control containing a mutated Rhi-milR-9829-5p targeting site did not affect luciferase expression levels (Fig. 7b). Consistent with the qRT-PCR results (Fig. 7e, g), these results indicate that *GRMZM2G412647*encoding a member of the Kelch motif family is a genuine target of Rhi-milR9829-5p.

To assess the involvement of GRMZM2G412674 in maize BLSB, we identified two maize ethyl methane sulfonate (EMS) mutant lines, *EMS3-001f31* (*f31*) and *EMS3-001f33* (*f33*), from the Maize EMS induced Mutant Database (MEMD) [48]. The *f31* and *f33* mutant line carried a nucleotide substitution at the site of 2150 bp and 1105 bp which caused an early stop codon and synonymous mutation, respectively (Fig. 7a). Interestingly, the expression levels of GRMZM2G412674 in *f31* and *f33* were reduced to 17 and 63% of that in the inbred line B73, respectively (Fig. 7c). The *R. solani* inoculation was performed in B73 and the two mutant lines in the field. We counted the disease index at 5 and 14 dpi and found that the *f31* and *f33* mutants exhibited more serious disease symptoms than B73 (Fig. 7f). The lesion length of *f31* and *f33* was increased by approximately 109 and 81% at 14 dpi compared to that of B73, respectively (Fig. 7d). Thus, these results demonstrate that GRMZM2G412674 act as a positive regulator of BLSB resistance.

## Discussion

A large number of miRNAs have been characterized in plants, animals and microorganisms [26, 27, 49–53]. Although it has been reported that Dicer-like Argonaute proteins exist in fungi and RNA silencing methods function as an antiviral defense mechanism [54, 55], the number of miRNAs identified in plant pathogenic fungi are not so numerous than that in plants and animals. One explanation is that the abundance of miRNAs is quite low or they are expressed only during specific stress stages, making them difficult to identify with traditional methods, such as microarray. Recently, with the development of high-throughput sequencing of small RNAs, it has become possible to generate large libraries of small RNAs to detect less abundant and novel miRNAs. In this study, hundreds of small RNAs were obtained, and 171 milRNAs of *R. solani* were identified using high-throughput Illumina technology. Using the *R. solani*-rice interaction system, Lin et al. [33] identified 177 fungal milRNAs during the infection. Unlike the rice system used previously [33], in this study, we used the *R. solani*-maize system to identify milRNAs in AG1-IA.Although some reads that are conserved between AG1-IA and maize might have been missed due to the elimination of IA-3d reads mapping to B73 genome, the described fungal milRNAs might be more accurate. Furthermore, different from the inoculation AG1-IA on rice leaves without plants [33], we inoculated the pathogen at the leaf sheath in living maize plants, which is much closer to their natural interaction. Therefore, the characterization of milRNAs in *R. solani* provides a useful workflow to predict more fungal milRNAs. However, further experimental approaches are needed to confirm the function of these milRNAs.

Target genes of the 171 milRNAs were predicted in AG1-IA, and their functional annotation revealed that the targets have roles in multiple pathways, such as MAPK and calcium, transport and hydrolysis. Moreover, milRNAs differentially expressed before and after infection were identified as 18 VA-milRNAs. In fungi, targets that function as CYPs, ABC transporters, CAZymes, secreted proteins and SCFs were included. Previous studies have demonstrated that these genes play important roles not only in growth and development but also in pathogenesis. It was reported that the regulatory roles of specific fungal milRNAs in plant immunity may lie in altering the expression of plant growth-associated genes rather than modulating plant immunity, allowing more energy or transient configurations to be devoted to defense or tolerance [56]. Therefore, milRNAs are important pathogenic factors that regulate growth and pathogenesis. In addition, based on the pathogenic PHI sub network, nine genes assigned to the PHI database were characterized, which could facilitate the understanding of pathogenic factors during the infection process at the systematic level.

In maize, 58 target genes were predicted as targets of 16 VA-milRNAs, and 28 genes were annotated in the B73 genome database. Among the target genes, one encoding an ADP-ribosylation factor (GRMZM2G357399) was identified, which functions during endomembrane trafficking and increases organ and seed size by promoting cell expansion [57, 58]. We also observed that the WD40 repeat family protein encoded by GRMZM2G353548 was regulated by fungal milRNA. WD40 proteins act in a variety of functions, ranging from signal transduction and transcription regulation to cell cycle control [59], all of which are pivotal for pathogenesis. Moreover, the transcription factor TCP18,which controls the fate of plant organ growth and regulates part of hormone biosynthesis and signal transduction pathways, was annotated [44, 45]. In addition, GRMZM2G025592 was annotated as a DNA methyl-transferase, which is involved in genomic stability during development and chromatin organization as well as the alteration of DNA methylation status in cold-stress quiescent cells [60, 61]. GRMZM2G887276, which encodes a MYB family transcription factor, was also annotated. Previous studies revealed that MYB transcription factors play various roles, including enhancement of abiotic resistance and regulation of the differentiation of transfer cells [62–64]. Particularly, the thaumatin-like protein (TLP), which plays a role in antifungal defense [65], was also characterized. Furthermore, GRMZM2G412674, which is a target of Rhi-milR-9829-5p, was validated to be a positive regulator of BLSB (Fig. 7f). This result supports that analysis of small RNA regulation between pathogen and host is a useful strategy to explore the regulators of BLSB. In addition, based on the correlation analysis of the expression patterns of VA-milRNAs and their putative targets, only 5 targets were tightly negatively with their VA-milRNAs, suggesting them as stronger candidates for successful cross-kingdom transfer and function in hosts. From this information, we inferred that the VA-milRNAs of AG1-IA may target maize genes during the infection process to interfere in host immunity. Notably, Rhi-milR-222a-5p and Rhi-milR-92a-3p have predicted target genes only in AG1IA rather than in maize, suggesting that these two milRNAs are fungal-specific regulatory factors.

*Cis*-element analysis of maize target genes could help to reveal the probable functions. After analyzing the *cis*-elements of the 58 target genes in maize, we found multiple *cis*-elements related to biotic and abiotic stress responses. Recent studies have revealed overlaps between biotic and abiotic stress [66], and miRNAs play crucial roles during this process [67, 68], indicating that the *cis*-elements responsive to abiotic stress may be involved in pathogenesis by regulating expression of the target genes. Notably, fungal elicitor responsive elements (Box-W1) were identified, suggesting that these genes might be involved in the response to fungal pathogens [12]. However, dissecting the molecular mechanisms involved in these processes requires furtherinvestigation.

## Conclusion

We obtained 137 conserved and 34 novel milRNAs of *R. solani* and characterized 18 VA-milRNAs during the infection process. Target genes of the 18 milRNAs both in fungi and maize were predicted, and functional annotation and GO analysis revealed their possible involvement in pathogenesis. Further, expression patterns of milRNAs during the *R. solani* infection process were detected by real-time PCR and correlated with the expression of both candidate targets in fungi and plants. Finally, we validated that the maize gene GRMZM2G412674 was decreased when co-transformed with Rhi-milR-9829-5p utilizing a dual-luciferase assay and it was a positive regulator of BLSB resistance confirmed by the inoculation of *R. solani* in the EMS mutants. These results showed a useful strategy to explore the regulators of BLSB in maize and AG1-IA. Overall, our study provides new insights for revealing the regulatory roles of milRNAs in *R.solani*-maize interactions and exploring possible pathogenic mechanisms of BLSB disease in maize.

## Methods

### Fungal strains and plant materials

The *R. solani* AG1-IA strain YWK196 was cultured on potato dextrose agar (PDA; 20% potato, 2% dextrose and 2% agar, w/v) at 28 °C. Maize (*Zea mays* L.) inbred line B73 was grown in the greenhouse at 22 °C and 60% relative humidity with a 16 h/8 h light/dark cycle. The third leaf sheath from the ground was inoculated withYWK196 and utilized for RNA extraction. Symptoms of B73 sheath at different stages after inoculation were shown in Fig. S2.To determine the lesion length of maize EMS mutants, eight plants of each line were inoculated and the lesion length was measured at five and seven days post inoculation, respectively. Independent experiments were repeated twice and significant analysis was performed.

### Small RNA library construction and high-throughput sequencing

Total RNA of AG1-IA, AG1-IA-3d and maize samples was extracted using TRNzol Universal Reagent (Tiangen, China). Small RNAs, ranging from 18 to 30 nt, were purified from 100 μg total RNA by a 15% TBE-urea denaturing polyacrylamide gel electrophoresis (PAGE) and ligated to specific adaptors at the 5′ and 3′ ends. After reverse transcription and amplification, products were sequenced on an Illumina GAII platform. Construction of the small RNA library and high-throughput sequencing were performed twice with two independent biological replicates, and the average value was calculated for further analysis. The raw sequencing data was submitted to the National Center for Biotechnology Information (NCBI) Sequence Read Archive (SRA) with the BioProject accession code PRJNA596921 corresponding to BioSample accessions SAMN13642263, SAMN13642264, SAMN13642265, SAMN13642266, SAMN13642267 and SAMN13642268.

### Identification of conserved and novel milRNAs

The raw reads were filtered to get the clean tags using in house perl process. After removing adaptor/acceptor sequences, adaptor-adaptor ligation contaminants, insert tags and low-quality tags, the retained clean reads were aligned against the reference genome by SOAP software to exclude the tags matching to exon-sense, intron-sense, exon-antisense and intron-antisense, respectively. Meanwhile, the clean reads were blast against all of the noncoding RNA families annotated in the Rfam (version 13.0) with default parameters [69] and aligned with all of the plants and fungal bunches in NCBI GenBank database (ftp://ftp.ncbi.nlm.nih.gov/genbank/) to eliminate rRNA, tRNA, snRNA, snoRNA and other small RNAs. The remaining small RNA reads were utilized for further analysis to identify conserved and novel milRNAs.

To identify candidate milRNAs of *R. solani* YWK196, miRDeep [70] and MIREAP (http://sourceforge.net/projects/mireap) softwares were used to find hairpin structures among the precursors of remaining small RNAs with the previously described criteria [71]. To reduce computation, expression of each tag was normalized to TPM, and reads with abundance under 2.5 TPM were excluded. Then, all candidate tags existing in both miRDeep and MIREAP were aligned to the miRBase database using BLASTN. Candidate milRNAs that were matched to sequences of miRBase with less than four mismatches were identified as candidate conserved milRNAs, while other milRNA tags were considered candidate novel milRNAs. Finally, the sequences of candidate conserved and novel milRNAs exactly matching the *R. solani* AG1-IA genome sequence (accession number AFRT00000000.1) but not the maize genome or cDNA were termed conserved and novel milRNAs, respectively, which were retained for further analysis.

### Target gene prediction of milRNAs

To predict potential target genes of milRNAs, psRNATarget online (http://plantgrn.noble.org/psRNATarget/analysis) [72] was used as previously described with default parameters. To predict target genes in AG1-IA and maize, milRNA sequences were aligned to The*r. solani* AG1-IA genome sequences and the B73 genome (http://www.maizegdb.org, release 5b+).

### Real-time RT-PCR of fungal milRNAs and target genes

To detect expression levels of fungal milRNAs, total RNA was isolated using TRNzol Universal Reagent (Tiangen, China) and treated with RNase-free DNaseI (Promega, USA) for the elimination of genomic DNA. Then, a miRNA cDNA Synthesis Kit (CWBio, China) was used for first strand cDNA synthesis with an oligo-dT adaptor. Real-time RT-PCR of milRNAs was performed using a miRNA qPCR Assay Kit (CWBio, China) with a forward primer for mature milRNA sequences and a universal reverse primer on a qTOWER3 touch real-time system (Analytik Jena AG, Germany). Expression of 18S rRNA gene was used as a normalization control. The threshold cycle (Ct value) was automatically recorded, and the ΔΔCt method [73] was used to calculate relative expression levels of milRNAs. The Rhi-18S rRNA gene was normalized as internal control in AG1-IA. Three replicates with three biological samples were performed for each experiment. Primers used in real-time PCR analysis are listed in Table S13.

Total RNA of IA, IA-3d and Maize were obtained by TRNzol Universal Reagent (Tiangen, China) and were then used to synthesize first strand cDNA with a PrimeScript RT Master Mix (Perfect Real Time) Kit (TaKaRa, Japan) according to the manufacturer’s protocol. Real-time RT-PCR was performed using a SYBR Premix Ex Taq (TliRNaseH Plus) Kit (TaKaRa). Rhi-18S rRNA and maize β-Tublin gene were normalized as internal control in fungi and maize, respectively. The ΔΔCt method was used to determine relative expression levels. All reactions were repeated three times with three biological samples. Primer sequences are listed in Table S14.

### Differential expression analysis of milRNAs

To represent normalized milRNA expression level, TPM was used and calculated with the following formula: actual milRNA count/total count of clean reads × 1000,000. For identification of milRNAs differentially expressed between IA-3d and IA, fold-change = log2(IA-3d/IA) was employed. Only milRNAs with fold-changes > 1.5 and *P*-values < 0.01 were selected as differentially expressed milRNAs for further analysis.

### GO analysis

GO analysis on target genes was performed by Singular enrichment analysis (SEA) (http://bioinfo.cau.edu.cn/agriGO/analysis.php) [74]. Gene terms in molecular functions, biological processes and cellular components categories were regarded as significantly enriched with *P-*value< 0.05.

### Statistical analysis

The correlation analysis between milRNA and target genes was performed by classical Pearson’s correlation tests as described previously using SPSS16.0 software [75] and a *P*-value < 0.05 was considered to be statistically significant. Meanwhile, the statistical analysis on real-time RT-PCR results was performed by one-way analysis of variance (ANOVA) followed by the Tukey method for pairwise multiple comparisons using Graphpad Prism 7.0 software. *P*-value < 0.05 was considered to be statistically significant and highlighted using different lowercase letters.

### Dual-luciferase reporter assay in tobacco leaves

To verify the predicted target of Rhi-milR-9829-5p (Supplementary Table S10), the 3’UTR sequence of GRMZM2G412674 was amplified from the cDNA of maize sheath and inserted into the pCAMBIA1300-LUC. To generate mature Rhi-milR9829-5p, the forward and reverse sequences of mature milRNA were synthesized by adding restriction sites (BamH I and Spe I for forward sequence; Sac I and Kpn I for reverse sequence) and ligated into the pCAMBIA1300–35-X vector. Then, the constructed Rhi-milRNA vector was cotransformed with the constructed vector of the target gene into *Nicotiana benthamiana* leaves. As a negative control, a mutated Rhi-milR-9829-5p targeting sequence (5′-TTCGTGTTGAGTCGGCATGCC-3′) of GRMZM2G412674 was designed and cotransformed with Rhi-milRNA after vector construction. The double reporter (firefly luciferase and Renilla luciferase genes) pGreenII0800-LUC vector was used as an internal control. Firefly and Renilla luciferase activities were quantified 72 h after infiltration with Dual-Luciferase Reporter Assay Systems (Promega) using Promega Glomax 2020 (Promega). Luciferase activity was assessed by the Firefly/Renilla (F/R) ratio, and three replicates with three biological samples were performed. Primers used are listed in Supplementary Table S15.

## Supplementary Information

**Additional file 1 Table S1.** List of conserved and novel milRNAs identified in *R. solani* AG1-IA.**Table S2.** Targets of milRNAs in *R. solani* AG1-IA.**Table S3.** Function annotation of target genes in *R. solani* AG1-IA.**Table S4.** List of target genes associated with pathogenesis. **Table S5.** GO analysis of target genes in *R. solani* AG1-IA.**Table S6.** milRNAs differentailly expressed between IA and IA-3d.**Table S7.** Target genes in *R. solani* AG1-IA of VA milRNAs. **Table S8.** Function annotation of target genes in *R. solani* AG1-IA of VA milRNAs. **Table S9.** GO annotation of target genes in *R. solani* AG1-IA of differentially expressed milRNAs. **Table S10.** Target genes in maize of VA milRNAs. **Table S11.** Function annotation and GO analysis of target genes in maize. **Table S12.** Number of *cis*-elements in the promoter of maize genes targeted by VA milRNAs. **Table S13.** Primers of milRNAs used for real-time PCR. **Table S14.** Primers of target genes in AG1-IA used for real-time RT-PCR.**Table S15.** Primers of target genes in maize used for real-time RT-PCR and 3′ UTR amplification.

**Additional file 2 Fig. S1.** Secondary structures of 10 milRNA precursors. **Fig. S2.** Symptoms of maize sheath inoculated by AG1-IA after (a) 0 h (b)12 h (c) 24 h (d) 3 days and (e) 5 days. **Fig. S3.** Features of plasmids used in the Luciferase assay. (a) Vector construction of mature Rhi-milR9829-5p using pCAMBIA1300–35-X. (b) Vector map of pCAMBIA1300-LUC used for the construction of GRMZM2G412674. (c) Vector map of the double reporter (firefly luciferase and Renilla luciferase genes) pGreenII0800-LUC vector which was used as an internal control.

## Data Availability

The data charts supporting the results and conclusions are included in the article and additional files. All sequences generated by sequencing for this study can be found in the NCBI Short Reads Archive (SRA) BioProject PRJNA596921 (https://www.ncbi.nlm.nih.gov/sra/?term=PRJNA596921) corresponding to BioSample accessions SAMN13642263, SAMN13642264, SAMN13642265, SAMN13642266, SAMN13642267 and SAMN13642268.

## Abbreviations

AG: Anastomosis group
BLSB: Banded leaf and sheath blight
DCL1: Dicer-like 1
LUC: Luciferase
PHI: Pathogen-host interaction
Pri-miRNAs: Primary miRNAs
RISC: RNA-induced silencing complex
miRNAs: MicroRNAs
milRNAs: microRNA-like small RNAs
nt: Nucleotides
TPM: Transcripts per million
PR: Pathogenesis related
ABC: ATP-binding cassette
TCDB: Transporter Classification Database
PHI: Pathogen-hose interactions
MAPK: Mitogen-activated protein kinase
TCP: Teosinte branched/Cycloidea/Proliferating cell factor.
CDC: Cell division cycle.
