# Supplementary figures and images for: Identification of virulence associated milRNAs and their bidirectional targets in Rhizoctonia solani and maize during infection
Source: BMC Plant Biol. 2021 Mar 26;21:155. doi: 10.1186/s12870-021-02930-w (PMC8004440; doi:10.1186/s12870-021-02930-w)

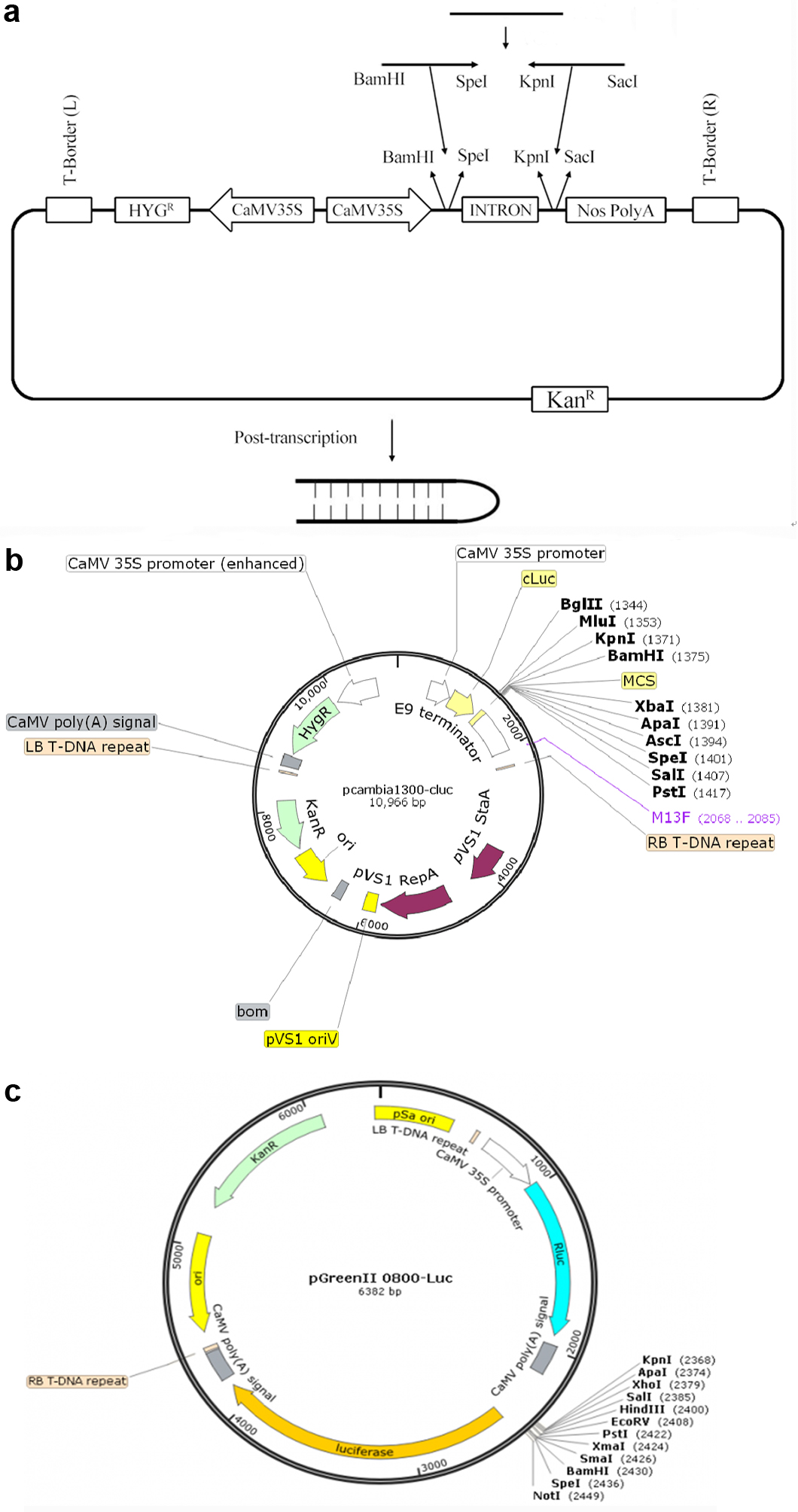

Supplement: Supplementary file 2 — Additional file 2 Fig. S1. Secondary structures of 10 milRNA precursors. Fig. S2. Symptoms of maize sheath inoculated by AG1-IA after (a) 0 h (b)12 h (c) 24 h (d) 3 days and (e) 5 days. Fig. S3. Features of plasmids used in the Luciferase assay. (a) Vector construction of mature Rhi-milR9829-5p using pCAMBIA1300–35-X. (b) Vector map of pCAMBIA1300-LUC used for the construction of GRMZM2G412674. (c) Vector map of the double reporter (firefly luciferase and Renilla luciferase genes) pGreenII0800-LUC vector which was used as an internal control. [file 12870_2021_2930_MOESM2_ESM.zip › 2.5 revised Figure S3.jpg]

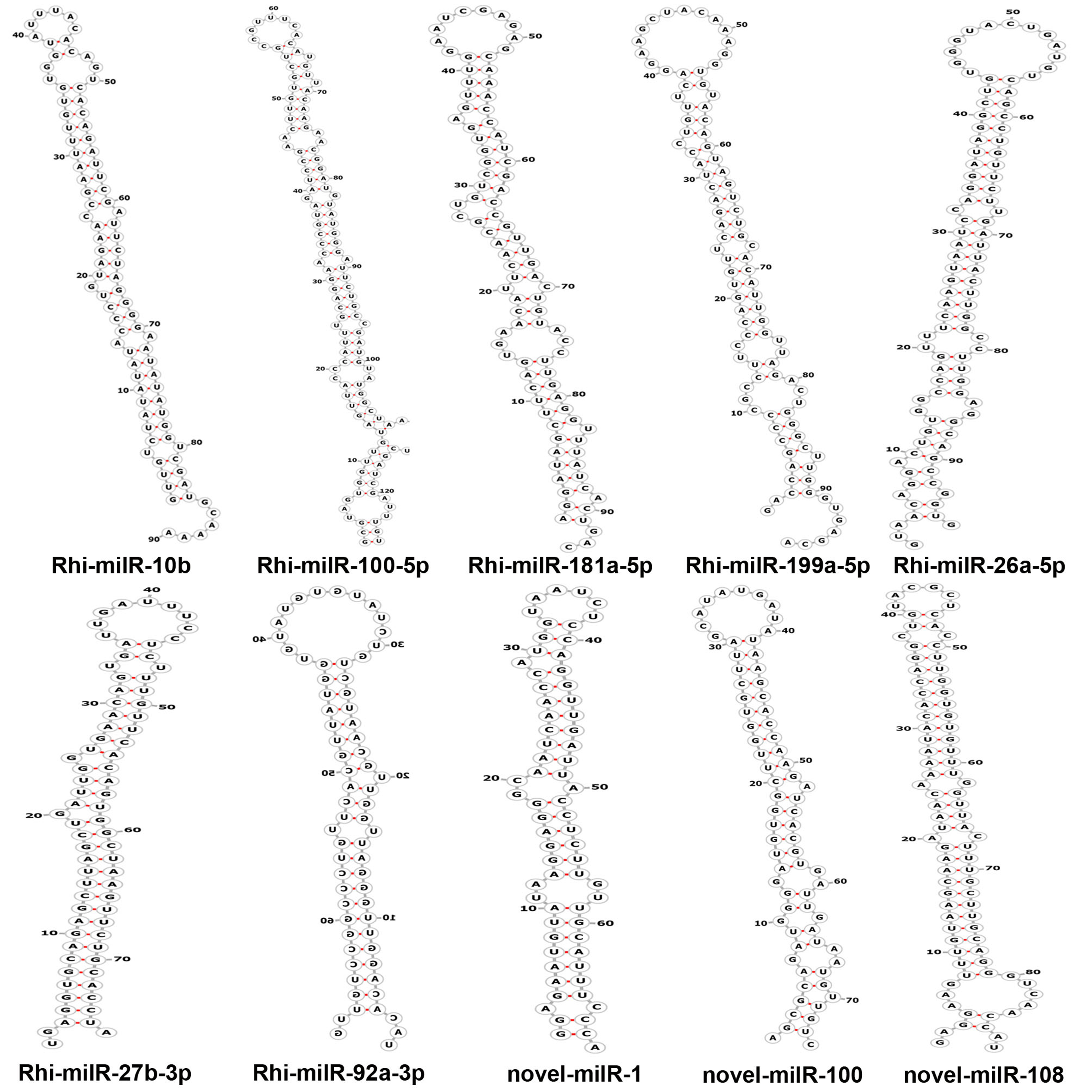

Supplement: Supplementary file 2 — Additional file 2 Fig. S1. Secondary structures of 10 milRNA precursors. Fig. S2. Symptoms of maize sheath inoculated by AG1-IA after (a) 0 h (b)12 h (c) 24 h (d) 3 days and (e) 5 days. Fig. S3. Features of plasmids used in the Luciferase assay. (a) Vector construction of mature Rhi-milR9829-5p using pCAMBIA1300–35-X. (b) Vector map of pCAMBIA1300-LUC used for the construction of GRMZM2G412674. (c) Vector map of the double reporter (firefly luciferase and Renilla luciferase genes) pGreenII0800-LUC vector which was used as an internal control. [file 12870_2021_2930_MOESM2_ESM.zip › Fig. S1.jpg]

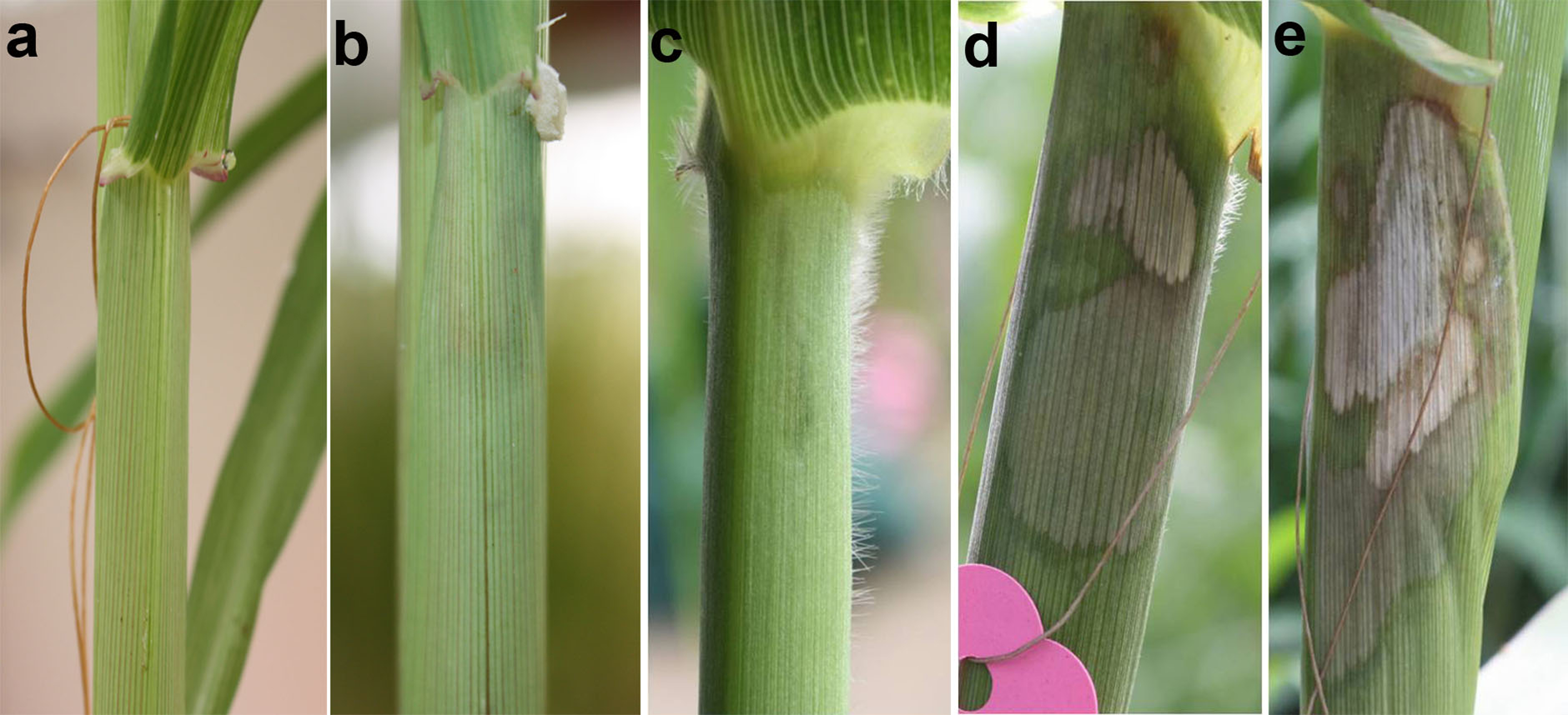

Supplement: Supplementary file 2 — Additional file 2 Fig. S1. Secondary structures of 10 milRNA precursors. Fig. S2. Symptoms of maize sheath inoculated by AG1-IA after (a) 0 h (b)12 h (c) 24 h (d) 3 days and (e) 5 days. Fig. S3. Features of plasmids used in the Luciferase assay. (a) Vector construction of mature Rhi-milR9829-5p using pCAMBIA1300–35-X. (b) Vector map of pCAMBIA1300-LUC used for the construction of GRMZM2G412674. (c) Vector map of the double reporter (firefly luciferase and Renilla luciferase genes) pGreenII0800-LUC vector which was used as an internal control. [file 12870_2021_2930_MOESM2_ESM.zip › Fig. S2.jpg]
